# The association between pre-gravid and first trimester maternal weight and its implications for clinical research studies

**DOI:** 10.1038/s41598-022-23510-4

**Published:** 2022-11-03

**Authors:** Ravi Retnakaran, Chang Ye, Shi Wu Wen, Hongzhuan Tan

**Affiliations:** 1 Leadership Sinai Centre for Diabetes, Mount Sinai Hospital, 60 Murray Street, Suite-L5-025, Mailbox-21, Toronto, ON M5T3L9 Canada; 2 Division of Endocrinology, University of Toronto, Toronto, ON Canada; 3 Lunenfeld-Tanenbaum Research Institute, Mount Sinai Hospital, Toronto, Canada; 4 OMNI Research Group, Department of Obstetrics and Gynecology, University of Ottawa, Ottawa, Canada; 5 Clinical Epidemiology Program, Ottawa Hospital Research Institute, Ottawa, Canada; 6 Department of Epidemiology and Community Medicine, University of Ottawa, Ottawa, Canada; 7 School of Public Health, Central South University, Changsha, China

**Keywords:** Outcomes research, Paediatric research

## Abstract

In clinical research, weight measurement in first trimester is often treated as a surrogate for pre-pregnancy weight. The validity of this critical assumption, however, is uncertain. Thus, we sought to prospectively evaluate the relationship between pre-gravid weight and first trimester weight. In this prospective preconception observational cohort study, 474 newly-married women in Liuyang, China, underwent pre-gravid evaluation at median 17.7 weeks before a singleton pregnancy, during which they had weight measurement in first trimester. The relationship between pre-gravid and first trimester weight was assessed by Bland–Altman analysis, Concordance Correlation Coefficient, and Pearson correlation. Mean pre-gravid weight was 49.8 ± 6.4 kg and mean weight in first trimester was 51.1 ± 7.0 kg. The Concordance Correlation Coefficient between pre-gravid and first trimester weight was 0.76 (95% limits of agreement: 0.72–0.80) and Pearson correlation was r = 0.78 (*p* < 0.0001), indicative of good concordance and correlation. As the timing of the weight measurement in first trimester increased in weekly increments from < 8 weeks to 14 weeks, the Concordance Correlation Coefficient ranged between 0.69 to 0.76 and the Pearson correlation ranged from 0.71 to 0.78 (all *p* < 0.0001). In conclusion, the observed concordance between pre-gravid weight and weight measured at any point in the first trimester provides a measure of validation for the widespread practice in clinical research of treating first trimester weight measurement as a surrogate for maternal weight before pregnancy.

## Introduction

In recent years, there has been growing recognition of the potential importance of optimizing maternal weight prior to conception for improving both obstetrical outcomes and long-term health of the offspring^1,2^. Indeed, a recent meta-analysis of individual participant data from 25 pooled cohort studies involving 196,670 participants showed that pre-pregnancy weight was generally more strongly associated with adverse obstetrical outcomes (preeclampsia/gestational hypertension, gestational diabetes, Caesarean delivery, preterm birth, and small-/large-for-gestational-age) than was gestational weight gain^3^. Moreover, with recognition of the Developmental Origins of Health and Disease (DOHaD) paradigm^4^, pre-gravid weight has emerged as a key maternal feature that potentially may affect the intrauterine environment and developmental programming at conception and beyond, thereby impacting long-term postnatal outcomes in the offspring.

Despite its physiologic and clinical importance, the ascertainment of pre-gravid weight is often problematic in research studies. Notably, for practical reasons, studies typically recruit women only after they are pregnant (i.e. because the cost of recruiting/characterizing non-gravid women and then waiting for pregnancy could be prohibitive within the limited time window of research funding). With recruitment in pregnancy, the opportunity for prospective measurement of pre-gravid weight is missed. Accordingly, a common practice in studies is to treat the weight measurement in first trimester as a surrogate for pre-pregnancy weight^5^. However, the validity of this critical assumption remains uncertain. Thus, our objective in this study was to prospectively evaluate the relationship between directly-measured pre-gravid weight and weight measurement in first trimester in the setting of a preconception cohort in which women were assessed prior to pregnancy and again in early gestation.

## Methods

### Cohort

In this prospective preconception cohort study, women were recruited at the time of marriage in the Liuyang region of Hunan province in China. The study protocol has been described in detail previously^6,7^. In brief, participating women were characterized at recruitment (pre-gravid) and then, upon a subsequent pregnancy, were followed across gestation through clinical care. This cohort study has been approved by the institutional research ethics boards of Central South University (Changsha, China), Ottawa Hospital Research Institute (Ottawa, Canada), and Mount Sinai Hospital (Toronto, Canada). The study was conducted in accordance with Good Clinical Practice and the principles of the Declaration of Helsinki. All participants provided written informed consent. The current analysis was restricted to women who had weight measurements at both baseline (pre-gravid) and within the first 14 weeks of gestation (n = 474).

### Exposure and outcome

As previously described^6,7^, the Liuyang Maternal and Infant Hospital was specifically selected for this preconception cohort because women in its catchment area (i) typically attend a pre-marriage health clinic assessment and (ii) tend to have a first pregnancy within the first year of marriage (based on societal practices in the region). Thus, by recruiting at the pre-marriage health clinics, we established a cohort of women who indicated that they planned to conceive in the next 6 months. Baseline assessment at recruitment included anthropometric measurements (waist, weight, height, calculated BMI) performed by trained research staff. Once pregnant, participants received obstetrical care at Liuyang Maternal and Infant Hospital, including weight measurements.

### Statistical analysis

All analyses were performed using SAS 9.4 (SAS Institute, Cary, NC). We used the interquartile range method to identify 14 outliers in either pre-gravid weight or first trimester weight. After examining the 14 data points and considering the large sample size of 474, we retained them in the data analysis. The relationship between pre-gravid weight and first trimester weight was evaluated in 3 ways. First, we performed Bland–Altman analyses^8^ to characterize the mean difference between these measurements. Second, we calculated the Concordance Correlation Coefficient^9^, which assesses agreement between two measures of the same continuous variable. Third, we evaluated the Concordance Correlation Coefficients and the Pearson correlations between pre-gravid weight and first trimester weight measurements performed at < 8 weeks, < 9 weeks, < 10 weeks, < 11 weeks, < 12 weeks, < 13 weeks, and < 14 weeks, respectively.

## Results

**Figure 1:**
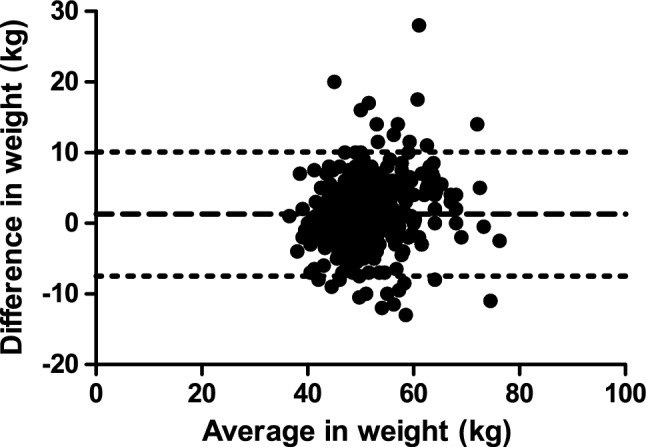
Bland–Altman plot of the difference between pre-gravid weight and 1st trimester weight measurement. Data shown as mean difference (dashed line) and 95% limits of agreement (dotted lines).

The study population consisted of 474 women who underwent baseline assessment at median 17.7 weeks before a singleton pregnancy (Table 1). Their mean pre-gravid weight was 49.8 ± 6.4 kg and mean weight in first trimester was 51.1 ± 7.0 kg. The Bland–Altman plot (Fig. 1) shows the bias and 95% limits of agreement of the mean difference between these measurements, revealing only a slightly positive bias. The Concordance Correlation Coefficient was 0.76 (95% limits of agreement: 0.72–0.80), indicating good concordance between pre-gravid and first trimester weight measurements. The Pearson correlation was r = 0.78 (*p* < 0.0001).

**Table 1:** Demographic and clinical characteristics of study population.

At pre-gravid assessment	N = 474
Weeks before pregnancy (weeks)	17.1 (5.3–46.9)
Age (years)	25.2 ± 3.1
Years of education (years)	9 (9–12)
Household income (1000 yuan)	20 (10–30)
Weight (kg)	49.8 ± 6.4
BMI (kg/m^2^)	20.1 ± 2.4
Waist (cm)	70.5 ± 7.5
Systolic blood pressure (mm Hg)	110.4 ± 12.6
Diastolic blood pressure (mm Hg)	70.7 ± 9.4
**In first trimester**	
Weight (kg)	51.1 ± 7.0
**At delivery**	
Length of gestation (weeks)	39 ± 1
Total gestational weight gain (kg)	16.6 ± 5.4

Continuous variables are presented as mean ± standard deviation (if normally distributed) or median followed by interquartile range in parentheses (if skewed).

We next sought to determine if there was a time point in the first trimester after which this relationship deteriorates. As shown in Table 2, neither the Concordance Correlation Coefficient nor the Pearson correlation between pre-gravid and first trimester weight declined appreciably as the timing of the latter measurement advanced by weekly increments from < 8 weeks to < 14 weeks. Specifically, the Concordance Correlation Coefficient ranged from 0.69 to 0.76 and the Pearson correlation ranged from 0.71 to 0.78 (all *p* < 0.0001). These data thus suggest that maternal weight measurement at any point in the first trimester can provide a reasonable surrogate for pre-gravid weight.

**Table 2:** Association between pre-gravid weight and first trimester weight in relation to the timing of the latter measurement within the first 14 weeks of gestation.

Timing of 1st trimester weight measurement ( weeks)	Number of women (n)	Percentage increase in weight from pre-gravid weight (mean ± SD)	Concordance Correlation Coefficient CCC (95% limits)	Pearson Correlation (r, *p*)
< 8	110	3.4 ± 10.6	0.69 (0.58, 0.77)	0.71, *p* < 0.0001
< 9	168	2.7 ± 9.9	0.70 (0.61, 0.77)	0.72, *p* < 0.0001
< 10	214	2.7 ± 10.0	0.72 (0.65, 0.78)	0.73, *p* < 0.0001
< 11	266	2.5 ± 9.5	0.75 (0.69, 0.80)	0.76, *p* < 0.0001
< 12	313	2.5 ± 9.2	0.76 (0.72, 0.81)	0.78, *p* < 0.0001
< 13	380	2.7 ± 9.1	0.77 (0.72, 0.80)	0.78, *p* < 0.0001
< 14	474	2.9 ± 9.2	0.76 (0.72, 0.80)	0.78, *p* < 0.0001

We also performed exploratory analyses in which these associations were evaluated in women with pre-gravid weight greater than and below the median in the study population (49 kg), respectively. In women with pre-gravid weight above the median, the percentage increase in weight from pre-gravid to first trimester ranged from 0.02 to 1.3% and the Concordance Correlation Coefficient ranged from 0.60 to 0.69 (data not shown). In contrast, in women with pre-gravid weight below the median, the percentage increase in weight ranged from 4.5 to 5.8% and the Concordance Correlation Coefficient ranged from 0.22 to 0.31 (data not shown), reflecting the comparatively greater impact of early pregnancy weight gain in leaner women.

## Discussion

In obstetrical research studies, the practical necessity of recruiting participants once they are pregnant means that there are limited options for retrospectively determining pre-gravid weight. These options include maternal self-report and inference of pre-gravid weight from measurements in pregnancy. However, each of these options has inherent limitations. Previous studies have documented that self-report of weight can be prone to under-estimation in young women^10,11^ A recent model for predicting pre-gravid weight from clinical parameters measured in pregnancy showed strong correlation with self-report but varied markedly in its performance between two study populations in which it was tested^12^. In this context, the simple approach of treating weight measurement in first trimester as a surrogate for pre-gravid weight has been widely adopted in research studies. This approach is based on the assumption that overall weight gain in the first trimester is modest in magnitude^5^. However, there has been limited evaluation of the validity of this practice, likely due to the paucity of studies with prospective recruitment prior to conception.

In an earlier study of 63 women, maternal weight at ~ 9 weeks gestation was 1.3 ± 3.0 kg higher than that which was measured before pregnancy, with a broad range in weight change observed between women (from − 5.2 to 13.5 kg)^5,13^. A recent study of 198 women who had a pregnancy within 3 months of recruitment in the Southampton Women’s Survey found that first-trimester weight measurement was 0.88 ± 2.34 kg higher than pre-pregnancy weight, again showing a broad range^14^. The current study supports the observation that weight gain in early pregnancy is generally modest but can vary, while extending this relationship across weekly increments from 8- to 14-weeks gestation and in a much larger population (n = 474). Moreover, in this prospective study, we have undertaken appropriate analyses (including Bland–Altman plot and Concordance Correlation Coefficient) for characterizing the relationship between pre-gravid and first trimester weight measurements. Recognizing that pre-gravid and first trimester weight measurements are unlikely to be identical (owing to the modest physiologic weight gain of first trimester), these analyses were reassuring in showing a slight positive bias (Fig. 1) but otherwise good concordance (Table 2). Accordingly, these data suggest that first trimester weight measurement can indeed provide a reasonable surrogate for pre-gravid weight, with the caveat that the difference between these measures is generally modest in magnitude but can vary.

Another caveat to note is that, though modest in magnitude, the early gestational weight gain from preconception to first trimester may hold specific physiologic effects, possibly by influencing the intrauterine environment and developmental programming (as per the DOHaD paradigm)^6^. Accordingly, modest weight gain in early pregnancy may be more strongly associated with outcomes that relate to such programming, as compared to greater weight gain later in gestation. For example, maternal weight before pregnancy and in the first 18 weeks of gestation, but not thereafter, has been shown to be a determinant of infant birthweight^6^. Thus, the suitability of first trimester weight as a surrogate for pre-gravid weight in research studies potentially may vary depending on the outcome of interest.

A unique strength of this preconception cohort is the prospective assessment of a large population of women at median 17.7 weeks before pregnancy. It is this feature that provided the capacity for characterizing the relationship between pre-gravid and first trimester weight measurements. We recognize that a limitation of this analysis is that the study population was comprised of a relatively homogenous subset of a single ethnicity (Chinese women with education level and household income shown in Table 1), such that these findings ideally require replication in other populations of different ethnicities, demographics and body habitus types. Furthermore, selection bias could arise from the decision to participate in this cohort. That said, the INTERGROWTH-21 project demonstrated that the pattern of weight gain in pregnancy is remarkably conserved in women across ethnic groups around the world (including China, India, Kenya, Oman, Brazil, Italy, United Kingdom and United States)^15^. Moreover, as described earlier, the setting of this study in Liuyang was specifically selected as one where features of the local environment (single maternity hospital and pre-marriage health assessment) made it possible to establish a large preconception cohort.

In conclusion, this prospective preconception cohort demonstrates that maternal weight in first trimester shows good concordance with pre-gravid measurement. Furthermore, the concordance between pre-gravid and first trimester weight is consistent across the first trimester from 8 to 14 weeks gestation. Thus, taken together, these data provide a degree of validation for the widespread practice in clinical research of treating first trimester weight measurement as a surrogate for maternal weight before pregnancy.

## Data Availability

De-identified data can be made available on request from the corresponding author upon appropriate institutional approval.
